# Social capital, policy fairness, and subjective life satisfaction of earthquake survivors in Wenchuan, China: a longitudinal study based on post-earthquake survey data

**DOI:** 10.1186/s12955-020-01594-8

**Published:** 2020-10-28

**Authors:** Dianxi Wang, Danyang Li

**Affiliations:** 1 Beijing Sport University, No. 48 Xinxi Road, Haidian District, Beijing, 100084 People’s Republic of China; 2 Beijing Police College, No. 11, Nanjian Road, Changping District, Beijing, 102202 People’s Republic of China

**Keywords:** Social capital, Policy fairness, Life satisfaction, Wenchuan earthquake

## Abstract

**Background:**

Existing research has rarely examined an earthquake’s sustained impact and the trajectory of the earthquake survivors’ life satisfaction over time. This study aims to analyze longitudinal changes in life satisfaction of Wenchuan earthquake survivors and the impact of social capital and government relief policy.

**Methods:**

This research applied a hierarchical linear model to longitudinal survey data collected after the earthquake. The survey was divided into three waves and was collected from Deyang City and Mianyang City of Sichuan Province. A random sampling method was used; a sample of 225 participants had valid responses over three waves of the survey.

**Results:**

This study found that a survivor’s social capital and the perception of the fairness of government relief policy have a significant effect on the trajectory of life satisfaction post-disaster. First, the initial life satisfaction of those with high social capital was significantly higher than of those with low social capital, whilst survivors with high social capital had a lower rate of change in life satisfaction. Second, one year after the earthquake, those who felt government policy was unfair had a lower life satisfaction than those who felt it was fair. However, from the first year to the fourth year after the disaster, survivors who believed that the government policy was unfair experienced a higher rate of change in life satisfaction than those who did not. Third, the fairness of government relief policy moderates the relationship between survivors’ social capital and changes in life satisfaction. A fair policy of relief can reduce the negative impact of the lack of individual social capital on life satisfaction.

**Conclusions:**

Survivors reconstruct life satisfaction through their social network and the perception of the fairness of the government’s post-disaster relief policy. Therefore, to promote the improvement of life satisfaction of survivors, it is necessary to cultivate social capital and ensure fairness of the relief policy.

## Background

On May 12, 2008, the Wenchuan earthquake occurred in Sichuan, China, leading to significant personal injuries and property loss. Even after a decade, people’s memories of this tragedy are still fresh. Entire cities and villages were destroyed; 7,789,100 houses collapsed, 69,227 people died, and 17,923 people disappeared [1], making this the most destructive earthquake since the reform and opening up of China. Although the earthquake occurred over 10 years ago, its effects can be seen even today. The earthquake’s continuous impact is reflected not only in the disaster area’s new geographical outline and village morphology but also in the multi-dimensional long-term effects on survivors’ psychological states, social relations, and quality of life (QOL). In particular, the Wenchuan earthquake may have also impacted people’s life satisfaction, which is a long-term impact; thus, this research focuses on changes in life satisfaction over an extended period of time post-disaster.

Many studies use survey data collected after natural disasters to analyze changes in people’s life satisfaction. The current consensus is that people’s life satisfaction drops sharply in the immediate wake of disaster and then slowly recovers. For example, Calvo et al. analyzed changes in the happiness of women affected by Hurricane Katrina and found that happiness significantly decreased between the pre-disaster period and the first year following the disaster, but there is no significant difference between pre-disaster happiness and current happiness [2]. Similarly, exposure to the Wenchuan earthquake was associated with multidimensional impairment in quality of life, including physical, psychological, and environmental domains [3], such as higher levels of post-traumatic stress disorder and lower levels of QOL than pre-earthquake [4, 5].

Regarding the impact mechanism of individuals’ life satisfaction after disaster, existing research has taken multiple directions. A review of existing literature shows that these influencing factors can be discussed within the framework of ecological systems theory, a perspective emphasizing that individual growth is nested within a series of mutually interacting environmental systems. These environmental systems can be divided into microscopic, mesoscopic, and macroscopic systems. The microscopic systems refer to the direct environment of individual activities, such as family and school; the interconnection between micro systems forms the mesoscopic systems; macroscopic systems refer to the social and cultural environment of individual activities [6, 7]. Based on the framework of ecological systems theory, we divide the systematic factors of life satisfaction after disaster into three levels: personal, community, and governmental.

At the *personal level*, scholars have focused on the relationship between a survivor’s personal characteristics and life satisfaction in a disaster environment. Liang et al., for example, found that five years after the Wenchuan earthquake, male survivors had higher health-related quality of life scores than women, and survivors with a higher level of education and monthly income had higher scores than those with a lower education and monthly income [8]. Valenti et al. also determined that 18 months after the 2009 L’Aquila earthquake in Italy, older and less-educated survivors scored lower on psychological quality of life [9]. Sugano, based on the data collected six months after the 2011 Great East Japan Earthquake, found that the life satisfaction of women survivors in their 60s and with high socioeconomic status decreased significantly [10]. Ardalan et al. assessed survivors’ QOL five years after the 2003 Bam earthquake in Iran and found that those living alone and those with poor living conditions had lower QOL [11].

Another research focus, also at the *personal level,* concerns the impact of social capital on life satisfaction changes after natural disasters. Relevant sociological literature relies on the social capital theory to argue that natural disasters destroy people’s social networks and create a need to reconstruct an individual’s trust. In a disaster environment, the loss of social capital and distortion of relationship networks may create psychological stress and social anxiety, ultimately leading to higher levels of social stress and lower levels of health and well-being [12, 13]. However, such research fails to recognize that earthquakes may also enhance individual social capital and lead to increased participation in community activities. Yamamura, for example, found that earthquakes have a significant positive effect on social capital investment for residents in the earthquake source area [14]. In addition, social capital was a key factor affecting the recovery of disaster survivors [15]. A rich stock of social capital can eliminate the adverse effects of disasters and reduce recovery time, by alleviating people’s psychological stress and improving the health and well-being of survivors [16]. Different types of social capital may have different effects on disaster recovery. A study on the psychological state of survivors of the Ya’an earthquake determined that structural social capital had a negative impact on depression; however, cognitive social capital had a positive impact on life satisfaction and mediated the negative impact of structural social capital [17].

At the *community level*, community reconstruction and emotional connection play important roles in people’s life satisfaction after disaster. During the post-disaster reconstruction process, the community as the basic unit of post-disaster reconstruction includes not only the construction of infrastructure, such as houses and roads, but also the restoration and reconstruction of community social capital, such as community emotions and community relations networks. In particular, the sense of community belonging and trust as measurement dimensions of community social capital are important aspects of post-disaster community reconstruction and have a major impact on improving post-disaster quality of life [18]. Community trust, a sense of belonging, and a network of relationships together serve as a substitute for formal institutions and markets [19] and can mitigate the impact of disasters on life satisfaction [20].

At the *governmental level*, government relief policy provides an important perspective in the analysis of changes in life satisfaction after disaster. Government policies for recovery significantly impact the psychological status and QOL of survivors [21, 22]. The fairness of government disaster relief is related to life satisfaction recovery after disaster, which concerns the fairness of relief resource allocation and social support. However, a household survey conducted after the Wenchuan earthquake showed that 20.32% (n = 1949) of survivors believed they were treated unfairly by government assistance after the earthquake [23]; this suggested that survivors do not fully trust governmental relief policy. In addition, the perceived fairness of government disaster relief is directly related to trust in the government, and trust in governmental work had significant effects on the subjective well-being of survivors and QOL [24, 25].

In sum, the existing literature suggests that the occurrence of natural disasters causes damage to people’s relationship networks, mental health, and QOL. However, there are gaps in the existing research. First, the sample data are usually cross-sectional and the time period between the earthquake and the assessment is usually brief. Little attention is paid to the changes in life satisfaction of survivors across different periods after the disaster. Therefore, this current research uses three-wave longitudinal survey data collected after the Wenchuan earthquake to analyze the changes in the trajectory of earthquake survivors’ life satisfaction. Second, social capital is an important mechanism affecting life satisfaction recovery after disaster. Rebuilding social capital is an important way to cope with post-disaster crises and psychological risks. Accordingly, this study emphasizes the critical role of social capital in helping individuals cope with disasters. Third, most of the research on post-earthquake subjective life satisfaction focuses on the impact of psychological barriers and post-traumatic stress disorder on the psychological dimensions of recovery, while leaving out the dimension of government post-disaster relief policy fairness. Such an analysis is vital, considering both that the government relief policy plays an important role in post-disaster reconstruction work and that the fairness of post-disaster policy is crucial to residents’ life satisfaction. The focus on the impact of government policy fairness on people’s life satisfaction after disaster reflects the novelty of this study. Therefore, based on above analysis, we propose the following hypotheses:

- *Hypothesis 1a: *At the personal level, the temporal change in life satisfaction after the Wenchuan earthquake is not completely consistent among different groups. That is, the life satisfaction after disaster of men, the highly educated, and married survivors is higher than that of women, the low-educated, and those unmarried in the three-phases survey.
- *Hypothesis 1b*: For the survivors of the Wenchuan earthquake, individual social capital and life satisfaction are significantly correlated. The trajectory of their life satisfaction over time will vary according to their level of social capital.
- *Hypothesis 2*: For survivors of the Wenchuan earthquake, community belonging and community trust will affect their life satisfaction and the changing trajectory of recovery post-disaster.
- *Hypothesis 3*: The fairness of government post-disaster relief policy will affect survivors’ life satisfaction and the trajectory of recovery post-disaster. Survivors who perceive the government’s post-disaster policy as fair have better life satisfaction than those who perceive it as unfair.

## Methods

### Data

The reconstruction of China’s Wenchuan area has received extensive international attention [26]. In order to understand the recovery process and developmental trajectory of individual life satisfaction in a disaster environment, this study uses data from a post-disaster social reconstruction longitudinal survey implemented in the earthquake-affected areas of Sichuan.

The longitudinal data used in this study were collected from Deyang City and Mianyang City of Sichuan Province, the most seriously affected areas. This survey has passed the ethical guidelines review of the Institutional Review Board of Tsinghua University and received written informed consent from all participants. First, 12 villages in Deyang City and Mianyang City were selected randomly. Second, the list of households in the village was taken as a sampling frame, and a total of 33 households were randomly selected from each village. Third, after entering the household, the Kish table was used to randomly select the respondents for investigation. The survey used a random sampling method and was divided into three waves. The baseline survey was collected in May 2009; a total of 558 questionnaires were distributed in 12 villages, and 466 valid questionnaires were collected, making the valid questionnaire returns-ratio 83.5%. The second follow-up survey was conducted in the same way in November 2010, and a total of 313 samples were followed collected in each of the 12 villages. In April 2012, the 12 villages were visited again, and a total of 225 questionnaires were collected. Therefore, the final valid traceable sample was 225 throughout the longitudinal survey. Based on the sample size, we performed power analysis with the two-sample means test method. Taking the variables of gender and life satisfaction after disaster as examples, the power value of wave 1 is 0.504, wave 2 is 0.658, and wave 3 is 0.501. The statistical power of our survey is greater than 0.5 but less than the desired power value of 0.8 as proposed by Cohen [27]. However, scholars recommend estimating the two-level hierarchical linear model (HLM); in the scenario of small sample sizes, restricted maximum likelihood estimation (RMLE) has shown potential to perform well [28]. Therefore, this study uses the RMLE method to estimate model parameters. We collected information on respondents’ demographic characteristics, family information, disaster damage, government assistance, and post-disaster recovery process. In addition, information on participants’ relationship networks, life satisfaction, and community-building involvement was gathered. This series of surveys has contributed information that is vital to understanding survivors’ psychological and life recovery trajectories and the social reconstruction process in areas affected by disaster.

### Measurements and variables

#### Dependent variable

The dependent variable in this study is life satisfaction after disaster, which we measured using multiple survey questions about the respondents’ subjective satisfaction with life. Regarding the measurement of life satisfaction, we referred to the Satisfaction with Life Scale (SWLS) proposed by Pavot and Diener [29]. Previous scholars have applied the SWLS in the Chinese scenario and found that this scale has good validity [30, 31]. We made some improvements in accordance with the circumstances of the Wenchuan earthquake disaster, mainly in the merger of the original 7-level scale into 4-levels (i.e., Strongly disagree, Disagree, Agree, Strongly agree). These items included: “In most ways my life is close to my ideal,” “The conditions of my life are excellent,” “I am satisfied with my life,” “So far I have gotten the important things I want in life,” and “If I could live my life over, I would change almost nothing.” The answers to these questions included four options: 1 = Strongly disagree, 2 = Disagree, 3 = Agree, 4 = Strongly agree. The Cronbach α coefficient of this scale is 0.722.

We obtained the total life satisfaction score by summing the scores for all questions. We then determined respondents’ subjective perceptions of life satisfaction after disaster in different waves of the survey. The distribution of the dependent variable is normal, and the minimum value of this variable is 5; the maximum value is 20.

#### Independent variable

The independent variables of this study include social capital and policy fairness. Social capital was measured by the following three variables: community belonging, community trust, and the “Bainian” network. The variables of community belonging and community trust measured community social capital, whilst “Bainian” network measured individual social capital.

Scholars suggest that in the Chinese context, an individual’s social network during the annual Spring Festival (the “Bainian” network) reflects the capacity and range of their overall personal relationship network and individual social capital [32, 33]. To this end, we measured the size of respondents’ “Bainian” network using the following three survey items: “The number of relatives who greet your family during the Spring Festival,” “The number of good friends who visit your family during the Spring Festival,” and “The number of other people who visit your family during the Spring Festival.” The Cronbach α coefficient of the scale is 0.698, and the Spearman correlation coefficients between different items in this scale are all greater than 0.4. We calculated the total score of “Bainian” network by summing scores for all three questions; this variable is skewed, and the natural logarithm is taken.

Community level variables include community belonging and community trust. The measurement of these two variables is based on the scale created by Chinese scholars Gui and Huang, which they designed according to actual circumstances in China [34], and which has been proved to have good validity. We measured community belonging with the following survey items: “Do you plan to continue living in the same village for the next few years?”; “Do you feel like you will live in this village for a long time?”; “Do you feel that your current village is suitable for living in?”; “Do you feel that living in your current village is very important to you?”; “Do you feel that living in your current village is very comfortable and homey?”; “Do you identify as a resident of this village?”; “Do you feel that your identity as a resident of this village is important to you?”; and “Do you feel like a member of the village?” Respondents were asked to respond according to a four-point scale: 1 = almost never, 2 = sometimes, 3 = often, 4 = almost always. The score of community belonging was established by calculating the sum of the above indicators; this variable is normally distributed. The Cronbach α coefficient of the scale is 0.929, and the Spearman correlation coefficients between different items in this scale are all more than 0.5.

The survey also included three questions to measure community trust: “To what degree do you trust your neighbors?”; “To what degree do you trust the village committee?”; and “To what degree do you trust those who reside in your community?” Respondents were given a four-point scale: 1 = no trust at allfully trust, 2 = little trust, 3 = somewhat trust, 4 = fully trust. The sum for these three questions was considered a respondent’s community trust score. The Cronbach α coefficient of the scale is 0.539, and the Spearman correlation coefficients between different items in this scale are all greater than 0.4. The distribution of community trust is skewed, and we also performed natural logarithm processing.

Finally, we measured respondents’ perceptions of policy fairness based on the question: “How fair do you think the government’s earthquake relief policy is?” Respondents answered using a four-category variable: 1 = very unfair, 2 = unfair, 3 = fair, 4 = very fair.

#### Control variables

Our control variables are gender, education level, and marital status. Gender is a dummy variable (0 = male, 1 = female). Educational level includes two categories: 0 = elementary school and below and 1 = above elementary school. Marital status is also a two-category variable: 0 = have a spouse and 1 = without spouse, including single, divorced and widowed.

### Statistical analysis

This study relied on the HLM for statistical analysis. The HLM can simultaneously study the influence of individual and community variables on the dependent variable and judge whether the relationship between these variables changes according to the individual’s social characteristics. The HLM can effectively overcome deficiencies in variance homogeneity, etc., thereby ensuring the accuracy of model parameter estimation and statistical inference [35].

In this study, we regarded the intra-individual differences between different survey time-points as the lower level of the data structure and the inter-individual differences as the upper level. We used the HLM statistical analysis method to establish five models, and the control variables and independent variables were successively incorporated into models 1–5.

Model 1 is an unconditional mean model, that is, a simple model consisting only of the repeated measurements of $Y_{ij}$ without other independent variables or the time-related parameter. This model can be used to divide the variance in the mean estimation of a dependent variable into its within- and between-individual, regardless of time [36]. The equations for this model are:

$$\begin{aligned} & {\text{Level } - \text{ 1}}\,{\text{model:}}\quad Y_{ij} = \pi_{0i} + \varepsilon_{ij} ,\quad \varepsilon_{ij} \sim N\left( {0,\sigma_{\varepsilon }^{2} } \right) \\ & {\text{Level } - \text{ 2}}\;{\text{model:}}\quad \pi_{{0{\text{i}}}} = \gamma_{00} + \zeta_{0i} ,\quad \zeta_{0i} \sim N\left( {0,\sigma_{0}^{2} } \right) \\ \end{aligned}$$

Model 2 is an unconditional growth model, consisting of the repeated measurements of $Y_{ij}$ with the time-related parameter. Level-1 only includes survey time-points (TIME) as an independent variable, and level-2 does not include any independent variables. This model can help estimate the rate of change without covariates. That is, it can characterize the change in life satisfaction at different survey time-points without adding any other variables. The equations for this model are:

$$\begin{aligned} {\text{Level } - \text{ 1}}\,{\text{model:}} & \quad Y_{ij} = \pi_{0i} + \pi_{1i} \left( {{\text{TIME}}_{ij} } \right) + \varepsilon_{ij} \\ {\text{Level } - \text{ 1}}\,{\text{model:}} & \quad \pi_{0i} = \gamma_{00} + \zeta_{0i} \\ & \quad \pi_{1i} = \gamma_{10} + \zeta_{1i} \\ \end{aligned}$$

Model 3, based on Model 2, further includes control variables (gender, education level, and marital status) and the independent variable of policy fairness to test the impact of policy fairness on changes in life satisfaction of survivors. Model 4 incorporates social capital variables on the basis of Model 2 to examine the influence of social capital on the trajectory of life satisfaction after disaster.

Finally, model 5 is the full model with all independent variables and the time-related parameter to analyze the effect of policy fairness and social capital on the longitudinal change of life satisfaction after disaster. The equations for the full models are:

$$\begin{aligned} & {\text{Level } - \text{ 1}}\,{\text{model:}}\quad Y_{ij} = \pi_{0i} + \pi_{1i} \left( {{\text{TIME}}_{ij} } \right) + \varepsilon_{ij} \\ & {\text{Level } - \text{ 2}}\,{\text{model:}} \\ & \pi_{0i} = \gamma_{00} + \gamma_{01} \left( {GENDER_{ij} } \right) + \gamma_{02} \left( {EDUCATION_{ij} } \right) + \gamma_{03} \left( {MARRIED_{ij} } \right) \\ & \quad + \,\gamma_{04} {\text{POLICY}}\_{\text{FAIRNESS}}_{i} + \gamma_{05} \left( {BAINIANNET_{i} } \right) + \gamma_{06} \left( {BELONGING_{i} } \right) \\ & \quad + \,\gamma_{07} \left( {TRUST_{i} } \right) + \zeta_{{0{\text{i}}}} \\ & \pi_{1i} = \gamma_{10} + \gamma_{11} \left( {GENDER_{ij} } \right) + \gamma_{12} \left( {EDUCATION_{ij} } \right) + \gamma_{13} \left( {MARRIED_{ij} } \right) \\ & \quad + \,\gamma_{14} {\text{POLICY}}_{{{\text{FAIRNESS}}_{i} }} + \gamma_{15} \left( {BAINIANNET_{i} } \right) + \gamma_{16} \left( {BELONGING_{i} } \right) \\ & \quad + \,\gamma_{17} \left( {TRUST_{i} } \right) + \zeta_{1i} \\ \end{aligned}$$

In Level 1 of the full model, survey time-points (TIME) explain the change in individuals’ life satisfaction at different times after the disaster. In Level 2, gender (GENDER), educational level (EDUCATION), and marital status (MARRIED) explain the differences in life satisfaction of individuals from different demographics after the disaster and set a cross-level random effect using perception of policy fairness (POLICY_FAIRNESS), the “Bainian” network (BAINIANNET), community belonging (BELONGING), and community trust (TRUST) to explain the differences in the change of the trajectory of life satisfaction of different individuals. In other words, this model analyzes how change in the trajectory of life satisfaction at different times varies based on perception of policy fairness and social capital. The parameter estimation uses the RMLE. In addition, we also defined high social capital (mean plus one standard deviation) and low social capital (mean minus one standard deviation) and substituted these values into the fitting model to obtain a typical fitted growth trajectory. We performed the more rigorous two-tailed test, and all data processing and statistical analysis were completed using the STATA 14.0 software.

## Results

### Descriptive statistics

Table 1 presents the basic distribution of the investigation sample. The proportion of men survivors is higher than that of women; survivors with a spouse are also higher than those without. There are more survivors with lower levels of education than those with higher levels.

**Table 1:** The sample distribution of three-waves survey (n = 225)

Wave 1		Wave 2		Wave 3	
Variables	n (%)	Variables	n (%)	Variables	n (%)
Gender		Gender		Gender	
Male	125 (55.56%)	Male	125 (55.56%)	Male	125 (55.56%)
Female	100 (44.44%)	Female	100 (44.44%)	Female	100 (44.44%)
Education level		Education level		Education level	
Elementary and below	146 (64.89%)	Elementary and below	146 (64.89%)	Elementary and below	142 (63.11%)
Above elementary	79 (35.11%)	Above elementary	79 (35.11%)	Above elementary	83 (36.89%)
Marital status		Marital status		Marital status	
Without spouse	22 (9.78%)	Without spouse	20 (8.89%)	Without spouse	29 (12.89%)
Have a spouse	203 (90.22%)	Have a spouse	205 (91.11%)	Have a spouse	196 (87.11%)

Table 2 shows the change of individuals’ life satisfaction after the earthquake. Survivors’ mean life satisfaction increased from 10.633 one year after the earthquake to 11.782 four years later. In general, it shows a linear increase in life satisfaction, indicating that people’s life satisfaction has gradually recovered after the disaster. The t-test shows that demographic differences (gender, education level, and marital status) in life satisfaction after disaster are not completely significant. We only observe difference between education levels at wave 1, and gender difference in life satisfaction after disaster at wave 3.

**Table 2:** Mean of subjective life satisfaction of survivors in different survey years

	Subjective life satisfaction after disaster					
	Wave 1	T-Test	Wave 2	T-Test	Wave 3	T-Test
Gender						
Male	10.628	− 0.032	11.496	0.685	12.128	2.41*
Female	10.64		11.26		11.35	
Education level						
Elementary and below	10.366	− 1.895*	11.308	− 0.658	11.726	− 0.471
Above elementary	11.09		11.544		11.886	
Marital status						
Without spouse	10.983	0.724	10.955	− 0.84	12.3	0.998
Have a spouse	10.582		11.438		11.732	
Mean of subjective life satisfaction	10.633		11.391		11.782	

^*^p < 0.05

### Social capital, policy fairness, and life satisfaction after disaster

To test the impact of social capital and policy fairness on individuals’ life satisfaction after disaster, we established five models (see Table 3).

**Table 3:** HLM model results of post-disaster subjective life satisfaction

	Parameter	Model 1	Model 2	Model 3	Model 4	Model 5
		B (SE)	B (SE)	B (SE)	B (SE)	B (SE)
Fixed effect						
Initial state $\pi_{0i}$						
Intercept	$\gamma_{00}$	11.435*** (0.120)	10.786*** (0.251)	11.267*** (2.015)	6.390* (2.756)	10.223*** (2.885)
Gender	$\gamma_{01}$			0.602 (0.490)	0.370 (0.511)	0.606 (0.493)
Education level	$\gamma_{02}$			0.798 (0.499)	0.658 (0.521)	0.678 (0.505)
Marital status	$\gamma_{03}$			0.263 (0.758)	− 0.161 (0.775)	0.230 (0.757)
Policy fairness	$\gamma_{04}$					
Fair				− 2.397* (1.149)		− 2.732* (1.162)
Unfair				− 3.355** (1.148)		− 3.737*** (1.157)
Very unfair				− 3.514** (1.190)		− 3.887*** (1.203)
“Bainian” net	$\gamma_{05}$				0.362 (0.571)	1.222* (0.580)
Community belonging	$\gamma_{06}$				0.085 (0.054)	− 0.015 (0.056)
Community trust	$\gamma_{07}$				0.004 (0.006)	0.005 (0.006)
Rate of change ${ }\pi_{1i}$						
Intercept	$\gamma_{10}$		0.324** (0.107)	0.490 (0.906)	2.256† (1.319)	0.453 (1.398)
Gender	$\gamma_{11}$			− 0.433* (0.213)	− 0.344 (0.216)	− 0.418* (0.213)
Education level	$\gamma_{12}$			− 0.250 (0.228)	− 0.194 (0.233)	− 0.205 (0.229)
Marital status	$\gamma_{13}$			− 0.127 (0.367)	− 0.067 (0.362)	− 0.137 (0.364)
Policy fairness	$\gamma_{14}$					
Fair				0.983* (0.498)		1.140* (0.503)
Unfair				1.114** (0.493)		1.315** (0.497)
Very unfair				1.129* (0.516)		1.328* (0.523)
“Bainian” net	$\gamma_{15}$				− 0.449 (0.274)	− 0.695* (0.277)
Community belonging	$\gamma_{16}$				− 0.012 (0.027)	0.028 (0.027)
Community trust	$\gamma_{17}$				0.001 (0.004)	− 0.001 (0.004)
Random effect/variance components						
Level 1						
Intra-individual variance	$\sigma_{\varepsilon }^{2}$	5.282 (0.352)	5.161 (0.344)	4.828 (0.325)	4.975 (0.333)	4.758 (0.322)
Level 2						
Initial state	$\sigma_{0}^{2}$	1.502 (0.329)	2.243 (1.104)	1.455 (0.811)	2.387 (0.409)	1.580 (0.851)
Rate of change	$\sigma_{1}^{2}$		0.016 (0.040)	0.001 (0.007)	0.031 (0.013)	0.015 (0.022)
Covariance	$\sigma_{01}$		− 0.191 (0.277)	− 0.027 (0.198)	− 0.275 (0.063)	− 0.091 (0.219)
Model testing						
Deviance		3177.878	3168.141	3092.493	3138.738	3075.766
AIC		3183.879	3180.142	3128.493	3174.739	3123.766
BIC		3197.423	3207.23	3209.624	3256.003	3231.941

Initial state represents life satisfaction at the time of the disaster and rate of change represents changes in life satisfaction within 4 years after the disaster

^†^*p* < 0.1, *p < 0.05, **p < 0.01, ***p < 0.001

At the personal level, according to the results of Model 5, the control variables (gender, education level, and marital status) had no significant effect on the initial state of survivors’ life satisfaction after disaster; these findings indicate no group differences in changes to life satisfaction after disaster. The difference of life satisfaction between survivors with differing amounts of social capital (“Bainian” network) in the initial state is 1.222. There is also a significant difference in the rate of change: the richness of an individual’s social capital significantly affects the change in the trajectory of their life satisfaction over time.

At the community level, the initial state and the rate of change of community social capital (community belonging and community trust) are not significant, and the impact of community social capital on life satisfaction after disaster needs further verification.

**Fig. 1:**
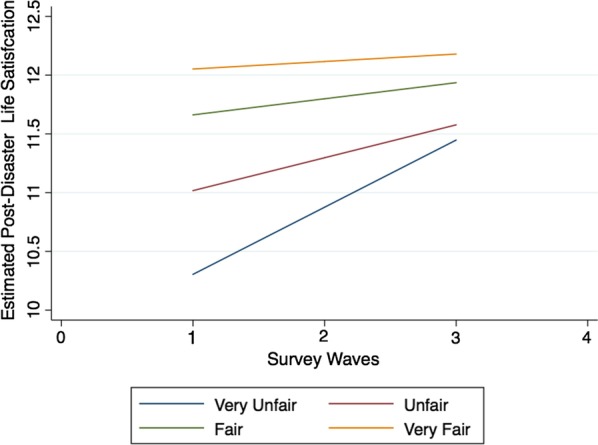
Fairness of relief policy and change in survivors’ subjective life satisfaction

At the governmental level, when other variables are controlled for, the initial state of life satisfaction of survivors who consider the post-disaster relief policy to be fair is higher than that of those who consider it unfair; there is also a significant difference in the rate of change. In other words, the differential perception of the relief policy’s fairness significantly affects the trajectory of survivors’ life satisfaction. The initial life satisfaction of survivors and its rate of change among those who feel that the government’s relief policy is fair is higher than that of survivors who feel that it is unfair (see Fig. 1). In addition, with time, the difference in life satisfaction between survivors who believe that the government’s relief policy is fair and survivors who believe that it is unfair is shrinking.

Random effects cause a reduction in the intra-individual variance ($\sigma_{\varepsilon }^{2}$) of Model 5 compared to Models 1–4, indicating that Model 5 explains more intra-individual variation. The independent variables included in Model 5 explain 30% of the intra-individual variation of life satisfaction, indicating that individual social capital and the fairness of the post-disaster relief policy are important predictors of the change in life satisfaction.

**Fig. 2:**
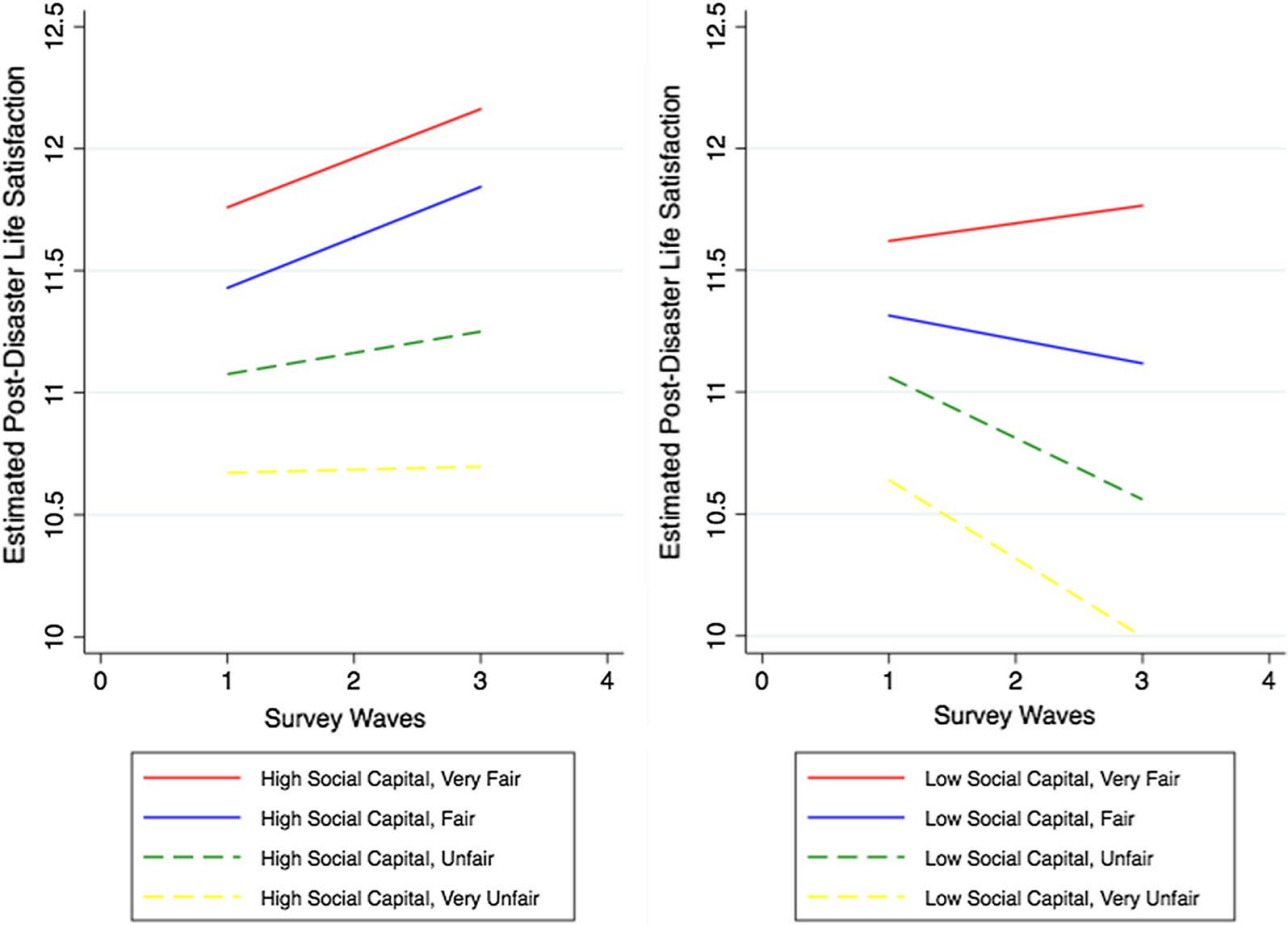
Social capital, fairness of relief policy and change in survivors’ subjective life satisfaction

Figure 2 presents the relationship between social capital, relief policy fairness, and changes in life satisfaction after disaster. We find that the fairness of government relief policy moderates the relationship between the survivors’ individual social capital and changes in life satisfaction. The unfairness of the policy can exacerbate the adverse impact of weak individual social capital on changes in life satisfaction, and the fairness of the policy can reduce the negative impact of the lack of individual social capital on changes in life satisfaction after earthquake.

In short, our descriptive statistical analysis shows that survivors’ life satisfaction after disaster gradually recovers over time. Our HLM models demonstrate that survivors’ individual social capital and their opinions regarding the fairness of government relief policy have a significant impact on the trajectory of their life satisfaction after the disaster. That is, the life satisfaction trajectory varies due to differences in perceptions of the government’s fairness and individual social capital.

## Discussion

The present study analyzes longitudinal survey data from the Wenchuan earthquake in China using an HLM model to determine temporal changes in survivors’ life satisfaction and the impact of social capital and the perception of the fairness of government relief policy. First, the demographic differences in life satisfaction after disaster are not completely significant, which does not fully support hypothesis 1a. Further testing is needed to determine whether demographic differences are statistically significant. Second, this study finds that the initial life satisfaction of survivors with high social capital is significantly higher than that of those with low social capital. Further, the rate of change is lower for those with high social capital than those with a low social capital. This finding supports hypothesis 1b and our theory regarding the impact of individual social capital on the trajectory of life satisfaction after disaster. Third, the effects of community belonging and community trust are not significant, and do not support hypothesis 2. Fourth, the initial life satisfaction of survivors who feel that the government’s relief policy is fair is higher than that of survivors who feel that it is unfair. The rate of change in life satisfaction is lower for those who feel that the policy is fair than for those who feel that it is unfair. This finding supports hypothesis 3. These findings indicate that survivors’ individual social capital and whether they believed the government’s disaster relief policy to be fair have a significant impact on the trajectory of life satisfaction after disaster.

Social capital is an essential factor of the residents’ life satisfaction and has a significant impact on families’ post-disaster recovery and increased life satisfaction [37]. As Hoogerbrugge and Burger noted, there is a significant correlation between social capital and residents’ life satisfaction [38]. Next, the improvement of trust and social connections can facilitate the reconstruction of families and communities affected by disasters [17, 37]. Social capital has an important impact on residents’ life satisfaction by enhancing social ties and cohesion; that is, families with more social capital recover more easily and faster, rebuilding resources through formal and informal networks of relationships [39, 40]. Survivors reconstruct their life satisfaction through individual social networks, thereby mitigating the negative impacts of earthquake damage and using their social networks to rebuild their lives to fit their idea of a good life [41]. In other words, in a disaster environment, rich social capital is conducive to the recovery and reconstruction of relationship networks after disaster, enabling survivors to recover from post-disaster trauma, rebuild their confidence in life, and continuously improve their life satisfaction after disaster.

In the post-disaster relief process, the fairness of disaster relief resource distribution and post-disaster reconstruction policy implemented by the government directly affects the life recovery process of survivors and their families, thus affecting survivors’ life satisfaction after disaster. The significant impact of the perception of government policy fairness on life satisfaction indicates that after a disaster, the government can serve as a source of hope, recovery, and stress relief. Survivors often pin their hopes on government policy, meaning that the survivors’ perception of the fairness of government relief policy affects the changes and structure of their life satisfaction after disaster [42]. Officials and policy makers should focus on the fairness of disaster relief policy. The fair design of post-disaster relief policy must pay attention to both the improvement of the material living standards and the reconstruction of the social networks of survivors [43].

Few studies have analyzed the life satisfaction of Wenchun earthquake survivors based on panel data, and the findings of this research can help to understand the trajectory and mechanism of survivors’ life satisfaction after disaster. The above findings suggest that in a disaster environment, social capital as a non-institutionalized social force has a strong explanatory power regarding individual life satisfaction. This can enhance our understanding of the relationship between individual social capital and disaster recovery, thus further enriching the knowledge on social capital. Furthermore, existing research has often ignored the impact of government relief policy on survivors’ life satisfaction; however, this study enhances the understanding of the role of policy factors in the changes of survivors’ life satisfaction after an earthquake. Specifically, the fairness of government relief policy moderates the relationship between survivors’ social capital and changes in life satisfaction, and a fair relief policy can reduce the negative impact of the lack of individual social capital on changes in life satisfaction.

Several limitations of this study must be considered. First, the survey locations were mainly the most severely affected areas. Therefore, the survey results may not fully represent the overall situation of life satisfaction of survivors in the entire Wenchuan earthquake-stricken area. However, this study presents a rare example of post-disaster longitudinal data, which is still of great significance for revealing the longitudinal changes and impact mechanism in life satisfaction of survivors of the Wenchuan earthquake. Second, this study only considers the changes in life satisfaction of survivors in the three years after the earthquake, and a detailed description with longer time period is not provided. When data are available, subsequent research can further analyze the change trajectory of life satisfaction over a longer time span. Third, the variable of policy fairness was measured only according to self-reported answers, and the Cronbach alpha for community trust score is low, which may have affected the validity of the measurement to a certain extent. Therefore, we recommend that future research use a comprehensive scale to measure these variables.

## Conclusion

This study finds that in a disaster environment, the strength of individual social capital and fairness of the government’s relief policy are predictors of changes in the survivors’ life satisfaction after disaster. At the individual level, survivors’ social capital can promote their life satisfaction after the disaster; the life satisfaction of the individuals with rich social capital can recover from the disaster environment more quickly. At the macro level, the fairness of the government’s relief policy also has a significant impact on the life satisfaction of survivors. A fair government disaster relief policy has a positive effect on the recovery of life satisfaction. This fact has certain implications for the formulation of post-disaster relief policy. On the one hand, the government should pay attention to the fairness of post-disaster relief and reconstruction policy. We need to ensure fairness and justice in the areas of relief force input, relief material distribution, post-disaster psychological support, reconstruction fund allocation, and infrastructure construction, thereby creating a favorable environment for survivors to recover from disaster and improve their satisfaction. On the other hand, social capital is also an important dimension of post-disaster reconstruction and should be fully utilized. It is necessary to restore the broken social network, and cultivate the relationship networks, which can provide emotional support for the improvement of life satisfaction of residents after the disaster.

## Data Availability

The datasets used and/or analyzed during the current study are available from the project of “Wenchuan Earthquake Longitudinal Survey” hosted by Tsinghua University.

## Abbreviations

HLM: Hierarchical linear model
QOL: Quality of life
RMLE: Restricted maximum likelihood estimation
SWLS: Satisfaction with life scale
